# Exploring the Potential Contribution of Climate-Informed Research to Future Ebola Preparedness in Central Africa

**DOI:** 10.3390/v18070782

**Published:** 2026-07-16

**Authors:** Sandra Ndaka Sumbu, Ben Bepouka

**Affiliations:** 1 Ministry of Public Health, National Communication Program for Health Promotion (PNCPS), Kinshasa P.O. Box 3088, Democratic Republic of the Congo; sandrandaka@gmail.com; 2 Office of Infectious Diseases and Global Health Research, University of Kinshasa, Kinshasa P.O. Box 123 Kinshasa XI, Democratic Republic of the Congo; 3 Infectious and Tropical Diseases Service, University Hospital of Kinshasa, University of Kinshasa, Kinshasa P.O. Box 123 Kinshasa XI, Democratic Republic of the Congo

**Keywords:** Ebola virus disease, climate-informed, preparedness, One Health, environmental monitoring, outbreak preparedness, Central Africa

## Abstract

The ongoing 2026 Bundibugyo ebolavirus outbreak in eastern Democratic Republic of the Congo highlights the continued vulnerability of Central Africa to recurrent Ebola emergence. This outbreak emerged less than six months after the previous one ended, appears to represent one of the shortest documented inter-epidemic intervals in the Democratic Republic of the Congo based on currently available outbreak reports. Current surveillance systems remain largely reactive, focusing on the detection of human cases after zoonotic spillover has occurred. While strengthening health systems, diagnostic capacity, and early case detection remains the cornerstone of Ebola preparedness, growing research suggests that environmental and climatic information may eventually contribute to a broader understanding of spillover risk within a One Health framework. However, current evidence remains insufficient to identify validated environmental indicators or operational thresholds capable of predicting Ebola spillover events. Recent modeling studies have demonstrated that environmental drivers of Ebola emergence remain highly context-dependent and cannot yet support operational early warning systems. This commentary argues that continued research integrating environmental monitoring, remote sensing, ecological observations, and epidemiological data may improve understanding of Ebola emergence and eventually contribute to future preparedness strategies. Rather than proposing climate-informed preparedness approaches as an operational prediction tool, we emphasize its potential as a complementary research priority requiring further validation before any operational implementation.

## 1. Introduction

Despite repeated Ebola outbreaks across Central Africa, preparedness systems remain largely reactive and centered on the detection of human cases after zoonotic spillover events have already occurred [1,2]. The ongoing 2026 Ebola virus disease (EVD) outbreak caused by Bundibugyo ebolavirus in eastern Democratic Republic of the Congo (DRC), mainly the provinces of Ituri, North Kivu and South Kivu with cross-border transmission to Uganda, once again highlights the persistent vulnerability of the region to recurrent Ebola emergence [3].

The exceptionally short interval between the two most recent outbreaks warrants scientific attention. Since the 1976 Yambuku outbreak, the Democratic Republic of the Congo has experienced numerous Ebola outbreaks, including the ongoing 2026 event [4,5]. The intervals between successive outbreaks have historically ranged from one to eighteen years [5,6]. The 2026 outbreak, however, emerged less than six months after the end of the Kasai outbreak in December 2025 [3,4]. Such rapid recurrence raises important questions about the ecological conditions associated with Ebola emergence rather than suggesting that outbreaks are isolated events separated by long periods of viral silence [7,8,9].

The World Health Organization (WHO) has reported that the outbreak is expanding in a context characterized by insecurity, population displacement, and operational challenges that complicate outbreak control [3]. Although substantial investments have strengthened outbreak detection, laboratory capacity, and emergency response over the past decade, most preparedness efforts continue to focus on responding to outbreaks after they have begun. Current surveillance systems remain predominantly reactive, requiring public health authorities to respond after human infections have already been detected.

This experience raises an important question: could future Ebola preparedness strategies benefit from better integration of environmental and climatic information?

Growing evidence suggests that ecological disruption, climate variability, land-use change, and human activities may influence ecological conditions associated with Ebola emergence. However, the magnitude, timing, and interaction of these factors remain insufficiently understood, and no validated environmental indicators currently exist for operational surveillance [7,8,10,11,12,13,14,15,16,17,18]. In this context, continued research integrating environmental monitoring, remote sensing, ecological observations, and climate-informed risk assessment may improve understanding of Ebola spillover ecology and eventually contribute to future preparedness strategies.

The 2026 Bundibugyo outbreak therefore offers an opportunity to reconsider how climate and environmental information might be integrated into future Ebola preparedness strategies across Central Africa.

Despite increasing interest in environmental determinants of zoonotic disease emergence, current scientific evidence remains insufficient to support reliable operational prediction of Ebola spillover events using environmental or climatic indicators alone. Recent modelling studies have shown that although ecological and climatic conditions may influence spillover risk, no consistent environmental thresholds or combinations of indicators have yet demonstrated sufficient predictive performance to guide public health decision-making. Consequently, climate-informed approaches should currently be viewed as complementary research priorities designed to improve understanding of Ebola ecology rather than as operational early warning tools [9,10,19,20].

## 2. From Outbreak Detection to Outbreak Anticipation

Most Ebola preparedness frameworks remain centered on outbreak detection and emergency response following the identification of suspected human cases. Clinical surveillance, laboratory confirmation, contact tracing, and case management continue to constitute the foundation of Ebola control and have substantially improved outbreak response capacity across Central Africa over the past decade.

Nevertheless, repeated Ebola outbreaks continue to highlight the importance of exploring complementary approaches that could enhance preparedness through a better understanding of the ecological conditions preceding zoonotic spillover. Within a One Health perspective, understanding the ecological and environmental contexts in which spillover events emerge remains an important scientific objective, although translating this knowledge into operational surveillance remains challenging.

Several studies have demonstrated that Ebola emergence is influenced by complex interactions among ecological, environmental, biological, and anthropogenic factors rather than by any single climatic variable. Forest fragmentation, land-use change, wildlife ecology, rainfall variability, and human activities have all been investigated as potential contributors to spillover risk, but their relative importance appears to vary considerably across locations and outbreaks.

Importantly, recent modelling studies have emphasized that these environmental relationships remain insufficiently consistent to support reliable operational prediction of Ebola spillover events. Telford et al. analysed more than two decades of documented Ebolavirus spillovers and concluded that currently available environmental data do not identify robust or actionable indicators suitable for operational early warning systems. Similarly, Baranowski and Bharti demonstrated that multiple environmental conditions may precede Ebola spillovers, but no reproducible combination of climatic or ecological variables consistently predicts spillover occurrence across Central Africa.

These findings underscore an important distinction between research-oriented environmental monitoring and operational public health surveillance. While environmental monitoring may improve understanding of spillover ecology and contribute to future research, current evidence does not support its use as a stand-alone predictive tool for Ebola preparedness.

Rather than proposing replacement of existing surveillance systems, we argue that future research should evaluate whether environmental observations—including remote sensing, ecological monitoring, climate information, and land-use indicators—can eventually complement existing surveillance systems once scientifically validated. Such research should proceed alongside continued investments in health-system strengthening, laboratory capacity, community surveillance, and rapid diagnostic capabilities, which remain the most effective and evidence-based strategies for reducing Ebola morbidity and mortality.

Consequently, the principal challenge is not to replace reactive surveillance with predictive systems that have not yet been validated, but rather to expand multidisciplinary research capable of improving understanding of the ecological conditions under which Ebola spillover occurs.

## 3. Climate and Environmental Drivers of Ebola Emergence

Although the ecological mechanisms underlying Ebola virus spillover remain incompletely understood, accumulating evidence suggests that environmental conditions may influence the ecological contexts in which spillover events occur. Climatic variability, forest fragmentation, land-use change, biodiversity loss, and changing patterns of human–wildlife interactions have all been investigated as potential contributors to Ebola emergence [7,8,9,10,11,12,19,20]. However, these factors are likely to interact in complex ways that differ across geographical settings and outbreak events rather than acting as universal predictors.

Fruit bats are widely considered the most plausible reservoir hosts for Ebolaviruses, although important uncertainties remain regarding reservoir ecology, viral maintenance in wildlife populations, and the precise pathways leading to human infection [11,12]. Environmental disturbances—including deforestation, mining activities, agricultural expansion, and increasing human encroachment into forest ecosystems—may modify wildlife habitats and increase opportunities for contact between humans and wildlife [2,8,10,11,12]. Nevertheless, direct causal relationships between specific environmental changes and individual Ebola spillover events remain difficult to demonstrate [9,19,20].

Several studies have reported associations between rainfall anomalies, vegetation dynamics, forest loss, and the spatial distribution of Ebola outbreaks [2,7,8,9,10]. Nevertheless, recent large-scale analyses have highlighted important limitations in translating these associations into operational surveillance tools. Telford et al. evaluated more than two decades of documented Ebolavirus spillover events across Africa and found that currently available environmental variables do not provide sufficiently robust or consistent signals to support predictive early warning systems [19]. Likewise, Baranowski and Bharti demonstrated that although multiple environmental conditions may precede spillover events, no reproducible combination of climatic or ecological indicators consistently predicts Ebola emergence across Central Africa [20].

These findings should not be interpreted as diminishing the importance of environmental research. Rather, they illustrate the complexity of Ebola spillover ecology and reinforce the need for multidisciplinary investigations integrating ecology, wildlife biology, epidemiology, environmental sciences, and public health [1,7,13,14,15,16,17,18,19,20]. Improved understanding of these interactions may ultimately contribute to more informative risk assessments, but current evidence does not yet support the operational use of environmental indicators as stand-alone predictors of Ebola outbreaks [19,20].

Within this context, environmental monitoring should currently be regarded primarily as a research-oriented component of Ebola preparedness research capable of improving scientific understanding of spillover ecology rather than as an operational early warning system [13,14,15,16,17,18,19,20].

## 4. The Limitations of Current Ebola Early Warning Systems

Current Ebola surveillance systems across Central Africa remain appropriately focused on the early detection of suspected human cases, laboratory confirmation, case investigation, contact tracing, and rapid outbreak response. These components have substantially improved Ebola control over the past decade and remain the cornerstone of outbreak preparedness [3,4,5,6]. Nevertheless, repeated outbreaks continue to expose important operational challenges, particularly in fragile settings characterized by insecurity, population displacement, limited healthcare access, and constrained diagnostic capacity [3,14,15,16,17,18].

Delayed recognition of the first human cases remains one of the major determinants of outbreak magnitude and duration. Previous studies have demonstrated that delayed diagnosis is consistently associated with larger and longer Ebola outbreaks, emphasizing the critical importance of strengthening health systems, community-based surveillance, laboratory capacity, and rapid diagnostic testing [21]. Consequently, investments in these evidence-based interventions should remain the highest priority for Ebola preparedness.

While environmental and climatic information may eventually improve understanding of the ecological contexts associated with spillover events, current scientific evidence does not support the routine operational integration of environmental indicators into routine Ebola early warning systems [19,20]. Existing studies have not identified reproducible environmental thresholds, spatial scales, or temporal indicators capable of reliably predicting spillover events across different ecological settings [19,20].

Accordingly, environmental monitoring should currently be viewed as a complementary research activity rather than as an operational surveillance strategy. Well-designed multidisciplinary prospective studies integrating environmental monitoring, ecological investigations, epidemiological surveillance, and advanced analytical methods may eventually identify more informative indicators of spillover risk. Until such evidence becomes available, strengthening existing surveillance systems and ensuring rapid detection of human infections remain the most effective public health interventions for reducing Ebola morbidity and mortality [21].

## 5. Toward Climate-Informed Ebola Preparedness

The integration of environmental and climatic information into Ebola preparedness should **not** be viewed as a replacement for existing surveillance systems. Rather, it should be considered a long-term research priority aimed at improving our understanding of the ecological conditions that may influence zoonotic spillover within a One Health framework [1,13,14,15,16,17,18,19,20].

Current Ebola preparedness continues to depend primarily on strong health systems, community-based surveillance, rapid case detection, laboratory confirmation, and effective outbreak response [3,4,5,6,21]. These interventions remain the best-supported evidence-based interventions that have consistently reduced outbreak size, duration, and mortality [21].

Although advances in satellite monitoring, remote sensing, environmental modeling, and geospatial analysis increasingly enable near real-time observation of environmental variables—including rainfall, vegetation dynamics, land-use change, forest loss, and temperature anomalies—current scientific evidence remains insufficient to determine which indicators, thresholds, spatial scales, or temporal patterns could reliably support operational Ebola early warning systems [13,19,20].

Consequently, environmental observations should currently be regarded as complementary sources of information for scientific investigation rather than independent operational triggers for public health action. Continued multidisciplinary research integrating ecological observations, wildlife surveillance, epidemiological data, and environmental monitoring may improve understanding of spillover ecology and eventually identify indicators that could strengthen preparedness strategies.

Within a One Health framework, future preparedness research may benefit from combining environmental information with epidemiological surveillance, wildlife monitoring, population mobility, and socioeconomic indicators to develop dynamic research-oriented risk assessment tools [13,14,15,16,17,18,19,20]. However, these approaches will require rigorous prospective validation before they can be incorporated into routine public health practice.

Artificial intelligence (AI) and machine-learning approaches may further facilitate the integration of heterogeneous datasets, including satellite observations, ecological monitoring, climatic variables, and epidemiological surveillance. Such approaches may help identify complex, non-linear relationships that conventional statistical methods may fail to detect. Nevertheless, AI should currently be considered an exploratory decision-support approach for research rather than a predictive solution. Any future AI-assisted research framework should undergo rigorous scientific validation, remain transparent and interpretable, and operate under continuous epidemiological oversight before informing public health decision-making.

**Figure 1:**
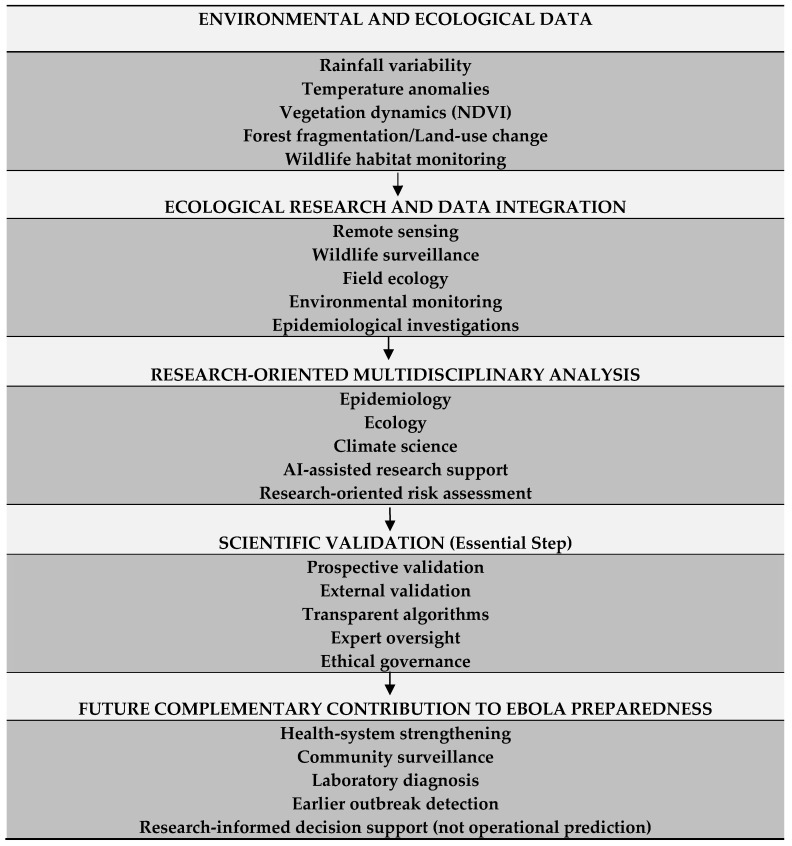
Conceptual framework for a research-oriented climate-informed preparedness framework for Ebola within a One Health approach. Environmental, climatic, ecological, and epidemiological data are integrated within a One Health framework to support multidisciplinary research on Ebola spillover ecology. Any future operational application would require rigorous prospective scientific validation, transparent analytical methods, and continuous public health oversight. This framework is presented as a research perspective rather than a validated operational early warning system.

Importantly, future climate-informed preparedness should complement—not compete with—investments in health-system strengthening, diagnostic capacity, workforce development, community engagement, and rapid response capabilities. These interventions remain the cornerstone of Ebola preparedness while research continues to improve understanding of environmental determinants of spillover. The proposed conceptual framework is illustrated in Figure 1. In addition, Table 1 summarizes the principal differences between current evidence-based Ebola preparedness and the proposed research-oriented climate-informed preparedness framework.

## 6. Limitations and Future Directions

This commentary is intended to stimulate discussion regarding future research priorities rather than to propose an operational framework for Ebola early warning. As a commentary, it does not present original experimental or epidemiological data, nor does it develop or validate predictive models for Ebola spillover. Instead, it synthesizes current knowledge and discusses future research directions at the interface of epidemiology, ecology, environmental sciences, and public health.

Current evidence indicates that environmental and climatic factors may influence the ecological context in which Ebola spillover occurs. However, the available literature also demonstrates that these relationships remain complex, heterogeneous, and insufficiently understood to identify robust environmental indicators suitable for operational surveillance [19,20]. No validated environmental thresholds, temporal patterns, or spatial indicators currently exist that can reliably predict Ebola spillover events across different ecological settings.

Accordingly, the concept of climate-informed preparedness presented in this commentary should be interpreted as a research perspective within a broader One Health framework rather than as an immediately deployable public health strategy. Future multidisciplinary studies integrating longitudinal ecological observations, wildlife surveillance, environmental monitoring, epidemiological investigations, remote sensing, and advanced analytical approaches will be necessary to determine whether environmental information can eventually complement existing surveillance systems.

Finally, any future application of climate-informed or artificial intelligence-assisted research should only be considered following rigorous prospective validation, transparent methodologies, interdisciplinary collaboration, and appropriate public health governance before being considered for operational implementation.

## 7. Conclusions

The 2026 Bundibugyo ebolavirus outbreak, occurring after an exceptionally short inter-epidemic interval, further highlights the continuing vulnerability of Central Africa to recurrent Ebola emergence and underscores the importance of strengthening preparedness strategies [3,4,5,6]. While the chronology of recent outbreaks raises important scientific questions regarding the ecological conditions associated with Ebola spillover, current evidence remains insufficient to support operational prediction of spillover events using environmental or climatic indicators alone [19,20].

Accordingly, strengthening health systems, community-based surveillance, laboratory capacity, rapid diagnostic testing, and timely outbreak response should remain the primary priorities for Ebola preparedness, as these interventions currently provide the strongest evidence for reducing outbreak size and mortality [3,4,5,6,21].

At the same time, continued multidisciplinary research integrating epidemiology, ecology, wildlife surveillance, environmental sciences, remote sensing, and climate observations within a One Health framework may improve understanding of the complex processes that influence Ebola emergence [1,7,8,9,10,11,12,13,14,15,16,17,18,19,20]. Such research could eventually identify environmental indicators capable of complementing existing surveillance systems, but substantial scientific validation will be required before any operational implementation can be recommended [19,20]. Emerging analytical approaches, including artificial intelligence and machine learning, may facilitate the integration of heterogeneous environmental and epidemiological datasets and support future research on dynamic risk assessment. However, these technologies should currently be regarded as complementary research-support tools requiring rigorous validation, transparency, expert oversight, and appropriate governance before informing public health practice [13,14,15,16,17,18,19,20]. Rather than advocating the immediate implementation of climate-informed Ebola preparedness strategies, this commentary proposes a multidisciplinary research agenda to investigate whether environmental and climatic information might eventually complement evidence-based Ebola preparedness within a One Health framework, provided that rigorous prospective validation demonstrates its added value before any operational application.

## Figures and Tables

**Table 1:** Comparison between current evidence-based Ebola preparedness and future climate-informed preparedness research within a One Health framework.

Component	Current Evidence-Based Ebola Preparedness	Research Framework for Future Climate-Informed Ebola Preparedness
**Primary objective**	Early detection and rapid response after identification of human cases	Improve understanding of spillover ecology (research only)
**Scientific evidence**	Strong evidence from multiple outbreaks	Emerging evidence; requires further validation
**Main data sources**	Clinical surveillance, laboratory confirmation, contact tracing, community surveillance	Climate observations, remote sensing, land-use change, wildlife surveillance, ecological monitoring, epidemiological data
**Analytical approaches**	Epidemiological investigation, laboratory diagnostics	Multidisciplinary analysis integrating epidemiology, ecology, environmental sciences, geospatial analysis, and AI-assisted research support
**Operational status**	Fully implemented and recommended by WHO	Research framework; not validated for operational use
**Current predictive capability**	Detects outbreaks after spillover	No validated environmental indicators currently available for reliable spillover prediction
**Current and future public health role**	Foundation of Ebola preparedness	Potential future complement to existing surveillance following scientific validation
**Current priority**	Strengthening health systems, laboratory capacity, rapid diagnostics, community surveillance	Multidisciplinary research to improve understanding of spillover ecology
**Key limitation**	Delayed recognition of index cases in some settings	Lack of validated environmental thresholds and prospective validation
**One Health contribution**	Coordination between human, animal and environmental health sectors	Expanded integration of ecological, climatic, wildlife and epidemiological information

Current Ebola preparedness relies primarily on health-system strengthening, laboratory confirmation, community surveillance, and rapid outbreak response. Climate-informed preparedness is presented as a research perspective within a One Health framework and should not be interpreted as a validated operational early warning system. Concepts summarized in this table are adapted from the literature on Ebola surveillance, One Health preparedness, and environmental determinants of zoonotic spillover [1,14,20,22].

## Data Availability

No new data were created or analyzed in this study. Data sharing is not applicable to this article.
